# Food Fingerprinting: LC-ESI-IM-QTOF-Based Identification of Blumeatin as a New Marker Metabolite for the Detection of *Origanum majorana* Admixtures to *O. onites*/*vulgare*

**DOI:** 10.3390/metabo13050673

**Published:** 2023-05-20

**Authors:** Marina Creydt, Friedemann Flügge, Robin Dammann, Burkhard Schütze, Ulrich L. Günther, Markus Fischer

**Affiliations:** 1 Institute of Food Chemistry, Hamburg School of Food Science, University of Hamburg, Grindelallee 117, 20146 Hamburg, Germany; robin.dammann@uni-hamburg.de; 2 Cluster of Excellence, Understanding Written Artefacts, University of Hamburg, Warburgstraße 26, 20354 Hamburg, Germany; 3 Institute of Chemistry and Metabolomics, University of Lübeck, Ratzeburger Allee 160, 23562 Lübeck, Germany; f.fluegge@uni-luebeck.de (F.F.); ulrich.guenther@uni-luebeck.de (U.L.G.); 4 LADR GmbH Medizinisches Versorgungszentrum Dr. Kramer & Kollegen, Lauenburger Straße 67, 21502 Geesthacht, Germany; b.schuetze@ladr.de

**Keywords:** metabolomics, food fraud, mass spectrometry, oregano, marjoram

## Abstract

Oregano (*Origanum vulgare* and *O. onites*) is one of the most frequently counterfeited herbs in the world and is diluted with the leaves of a wide variety of plants. In addition to olive leaves, marjoram (*O. majorana*) is often used for this purpose in order to achieve a higher profit. However, apart from arbutin, no marker metabolites are known to reliably detect marjoram admixtures in oregano batches at low concentrations. In addition, arbutin is relatively widespread in the plant kingdom, which is why it is of great relevance to look for further marker metabolites in order to secure the analysis accordingly. Therefore, the aim of the present study was to use a metabolomics-based approach to identify additional marker metabolites with the aid of an ion mobility mass spectrometry instrument. The focus of the analysis was on the detection of non-polar metabolites, as this study was preceded by nuclear magnetic resonance spectroscopic investigations of the same samples based mainly on the detection of polar analytes. Using the MS-based approach, numerous marjoram specific features could be detected in admixtures of marjoram >10% in oregano. However, only one feature was detectable in admixtures of >5% marjoram. This feature was identified as blumeatin, which belongs to the class of flavonoid compounds. Initially, blumeatin was identified based on MS/MS spectra and collision cross section values using a database search. In addition, the identification of blumeatin was confirmed by a reference standard. Moreover, dried leaves of olive, myrtle, thyme, sage and peppermint, which are also known to be used to adulterate oregano, were measured. Blumeatin could not be detected in these plants, so this substance can be considered as an excellent marker compound for the detection of marjoram admixtures.

## 1. Introduction

The dried leaves of oregano (*O. vulgare* and *O. onites*) are mainly used for flavoring food. Especially popular is the use of oregano as a spice in Mediterranean cuisine. However, oregano is one of the most counterfeited herbs in the world and is particularly often diluted with foreign plant material. There are several guidelines for trading oregano as food, to prevent such practices: According to the ISO standard 7925:2015, all *Origanum* genera can be sold as oregano with the exception of *O. majorana* [1]. Additionally, the Codex Alimentarius Committee on Spices and Culinary Herbs has specified that all *Origanum* genera may be used as oregano herbs, with the exception of *O. majorana*, too. The proportion of foreign matter must not exceed 0.1% [2]. According to the European Spice Association (ESA), only *O. vulgare* and *O. onites* may be traded as oregano, and the percentage of foreign substances must not exceed 2% [3,4].

The proportion of fake oregano samples on the market was recently published by the European Commission in its report: “Results of an EU wide coordinated control plan to establish the prevalence of fraudulent practices in the marketing of herbs and spices” from the year 2021. According to these studies, 48% of the oregano samples examined were conspicuous, largely because they were probably intentionally stretched with foreign plant material such as olive leaves, marjoram and myrtle. A whole range of different analytical methods were used to detect such falsifications. These included DNA-based methods, Fourier transform infrared spectroscopy (FTIR) to detect foreign matter based on outliers in combination with principal component analysis (PCA), X-ray fluorescence (XRF) spectroscopy for the detection of olive leaves, in particular based on copper content, and analyses of oleuropein, also a marker substance for olive leaves, using high performance liquid chromatography-high resolution mass spectrometry (HPLC-HRMS) [5].

DNA-based methods are increasingly used for the detection of foreign plant material because they are very sensitive [6,7]. However, genetic screening methods have the disadvantage that they are not optimally suited for quantification, first, because the quantity of DNA in plants can vary and, second, the DNA recovery rate is not constant during extraction, so that the amount of DNA does not correlate with the foreign plant content in a mixture. For this reason, ESA recommends in a white paper that qualitative DNA approaches should be combined with other analytical methods. These include the use of microscopic methods, which, however, also often depend on the operator and experience, as well as the use of non-targeted methods such as nuclear magnetic resonance (NMR) spectroscopy, near-infrared (NIR) spectroscopy, mid-infrared (MIR) spectroscopy and mass spectrometry (MS) [8].

Accordingly, various analytical approaches have been developed in this context in recent years, which also relate to the detection of the authenticity of oregano. These include methods based on NMR spectroscopy, which we recently used on oregano and marjoram samples, to detect different species, origins and various adulterations [9,10]. Some approaches have also been taken with MS analyzers: Thus, Black et al. and Wielogorska et al. first applied a non-targeted approach using a liquid chromatography instrument coupled to an electrospray ionization quadrupole time-of-flight-mass spectrometer (LC-ESI-QTOF) to identify marker molecules for plant impurities in oregano mixtures, and later developed a simpler targeted method for a liquid chromatography electrospray ionization triple quadrupole mass spectrometer (LC-ESI-QQQ) [11,12]. In addition to such classical LC-ESI-MS platforms, ambient MS techniques with direct analysis in real time (DART) sources or atmospheric solids analysis probe (ASAP) units have also been employed to achieve a corresponding reduction in sample preparation and measurement times [13,14]. Other approaches are based on various spectroscopic methods such as FTIR and NIR, which are generally not quite as sensitive as MS- or NMR-based methods but are very suitable for a quick and easy low-cost analytical verification [11,12]. These approaches might be sufficient considering that, according to the European Commission report, comparatively high levels (10–50%) of foreign plants are often added to oregano to maximize profit [5]. Van de Steene et al. recently published a study using five different analytical technologies to prove the authenticity and provenance of oregano. These included different mass spectrometric techniques as well as various spectroscopic strategies [15]. In the studies published to date, the main focus was on detecting the following plant admixtures (leaves): olives (*Olea europaea*), myrtle (*Myrtus communis*), cistus (*Cistus incanus*, *C. cyprius*, *C. creticus*), sumac (*Rhus coriaria*), hazelnuts (*Corylus avellana*), rhododendron (*Rhododendron indicum*), thyme (*Thymus serpyllum*), sage (*Salvia officinalis*) and phlomis (*Phlomis cytherea*) [9,10,11,12,13,14,15]. The European Commission report on the counterfeiting practices of oregano indicated that olive leaves were added to most samples, followed by *O. majorana* and myrtle leaves. In addition, a DNA-based approach detected greater proportions of *T. vulgaris*, peppermint (*Mentha piperita*) and *Saliva* spp. Due to the close phylogenetic relationship of the latter three plant species with *Origanum*, these could be false positive detections. However, a relatively high number of DNA reads were detected in some of the samples, which is why it is assumed that these could also be adulterations [5].

**Figure 1:**
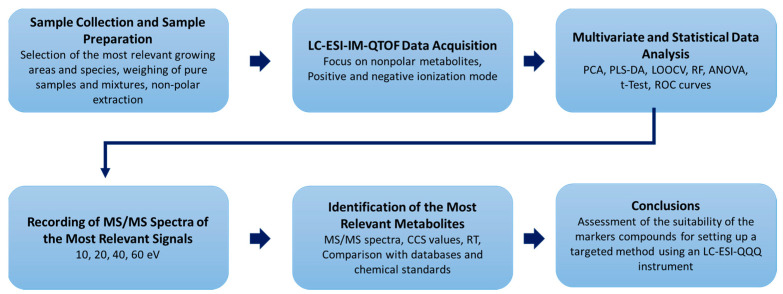
Schematic workflow of the presented study. Abbreviations: ANOVA, analysis of variance; CCS, collision cross section; LOOCV, leave-one-out cross-validation; PCA, principal component analysis; PLS-DA, partial least squares data analysis; ROC, receiver operating characteristic; RF, random forest; RT, retention time.

Although numerous studies have now been published and adulterations with *O. majorana* is reported to be of great importance, to our knowledge there are only two studies that have addressed the quantitation *of O. majorana* in oregano samples. One study was based on an ASAP-MS platform, which allowed the detection of *O. majorana* in *O. vulgare* samples only at proportions >50% [13]. The other study was recently published by us, in which we used an NMR-based approach, which allowed to detect additions of *O. majorana* at approx. 5%. In this context, in particular, arbutin turned out to be a suitable marker compound [10]. Since the content of arbutin in marjoram can vary greatly according to the literature, even if we could not confirm this in our own studies, and arbutin occurs relatively frequently in the plant kingdom, the aim of the present study was to identify further marker compounds with which admixtures of *O. majorana* in oregano samples can be detected [16,17,18]. A liquid chromatography electrospray ionization ion mobility quadrupole time-of-flight mass spectrometer (LC-ESI-IM-QTOF) was used to identify small organic marker compounds (*m*/*z* < 1500), suitable for detecting marjoram admixtures in oregano samples. A relatively non-polar extraction and chromatographic procedure was chosen, as similar analyses of food have been shown to be particularly useful for lipidomics approaches [19,20,21]. In addition, a different analytical window was covered compared to the NMR-based analyzes that preceded this study, thus increasing the overall probability of identifying further marker compounds. The analytical workflow performed is shown in Figure 1.

## 2. Materials and Methods

### 2.1. Chemicals

Acetonitrile, isopropanol, methanol (all LC-MS grade) as well as chloroform (HPLC grade), and ammonium formate (≥95% puriss.) were purchased from Carl Roth GmbH (Karlsruhe, Germany). Acetic acid (≥99.5% p.a.) was bought from Merck KGaA (Darmstadt, Germany). Ultrapure water (18 MΩ cm) was obtained from a millipore water purification system (Direct-Q 3 UV-R system, Merck KGaA, Darmstadt, Germany). Hexakis(1H,1H,3H-perfluoropropoxy)phosphazene, purine and LC/MS calibration standard for ESI-TOF were purchased from Agilent Technologies (Santa Clara, CA, USA). Hesperetin reference standard was obtained from Sigma-Aldrich (Steinheim, Germany), blumeatin reference standard from Cayman Chemical (Ann Arbor, MI, USA) and divanillin reference standard from Sigma-Aldrich.

### 2.2. Samples

In total, 39 oregano and marjoram samples were analyzed. These included 14 *O. onites*/*vulgare* samples from Turkey (TR), 10 *O. vulgare* samples from Greece (GR), 10 *O. majorana* samples from Egypt (EG), and 5 *O. vulgare* samples from Chile (CL). The dried and grounded samples were provided by or purchased from various European suppliers. All samples were genetically characterized using a DNA-based method as previously described [10]. However, DNA analysis revealed that the samples from Chile, which according to the declaration were supposed to be pure oregano samples, also contained relatively high levels of *O. majorana* in addition to *O. vulgare*. Nevertheless, these samples were also measured, but they were not included in the following mixing experiments. To identify suitable marker compounds that allow the detection of *O. majorana* in mixtures, blends of three *O. onites/vulgare* (TR) and *O. vulgare* (GR) samples were prepared with *O. majorana* (EG). The following ratios were evaluated: 95/5; 90/10; 85/15; 80/20; 75/25; 70/30; 50/50.

To determine whether the marker compounds for marjoram are also present in other plants used for adulteration, three dried samples each of *O. europaea*, *T. serpyllum* and *T. vulgaris*, *Saliva* ssp. (*S. officinalis* and *S. apiana*), *M. piperita* as well as *M. communis* were measured.

### 2.3. Sample Extraction

For the extraction of the non-polar analytes, a modified protocol from Bligh and Dyer was used, with which convincing results had already been obtained in previous studies on comparable matrices [22,23]. A total of 50 mg of each sample was weighed and 750 µL of a mixture of chloroform and methanol (1:2, *v*/*v*) was added. The samples were homogenized for 1 min at 3 m/s using two steel balls (3 mm in diameter) and a ball mill (Bead Ruptor 24, Omni International IM, GA, USA). Subsequently, 250 mL of chloroform and 500 mL of water were added, and the samples were crushed again with the ball mill for a further 2 min. The extracts were centrifuged at 18,000 g and 4 °C for 20 min (Centrifuge 5430 R, Eppendorf, Hamburg, Germany). Then, 100 µL of the lower phase was taken and diluted with 900 µL of isopropanol. The samples were centrifuged again and 500 µL of the liquid supernatant was transferred to a glass vial (Macherey-Nagel GmbH & Co. KG, Düren, Germany). To avoid changes of the analytes during preparation, all steps were carried out under ice cooling and with ice-cold solvents.

### 2.4. LC-ESI-IM-QTOF Analysis

Sample extracts were analyzed using an UHPLC system (1290 Infinity II, Agilent Technologies) coupled to an Agilent 6560 IMQTOF-MS instrument (Agilent Technologies), equipped with an ESI source (Dual JetStream, Agilent Technologies) and a gas kit (Alternate Gas Kit, Agilent Technologies). Chromatographic separation was carried out on a reversed phase C18 column (1.7 µm, 150 × 2.1 mm, Phenomenex, Aschaffenburg, Germany). The column was maintained at 50 °C during measurements and the flow rate was 0.250 mL/min. The mobile phases consisted of water (A) and isopropanol/acetonitrile (3:1, *v*/*v*) (B). For measurements in positive ionization mode, 0.1 mmol/L ammonium formate was added to the solvents. Measurements in the negative ionization mode were performed with 0.02% acetic acid in the solvents. The gradient used for both methods was set as follows: 0.0–2.0 min (55% B); 2.0–4.0 min (55–80% B); 4.0–22.0 min (80–100% B); 22.0–23.0 min (100% B); 23.0–24.0 min (100–55% B); 24.0–27.0 min (55% B). For the analysis in positive ionization mode 8 µL of the extract were used and for the measurements in negative ionization mode 10 µL. The autosampler temperature was kept at 5 °C.

Measurements were first carried out in positive ionization mode and then in negative ionization mode. In both modes, a mass range of *m*/*z* 50–1700 was recorded, which was calibrated directly before the measurements using the Agilent Technologies ESI tune mix. The ESI settings were as follows: gas temperature 300 °C; drying gas flow rate 12 L/min; nebulizer 35 psi; sheath gas temperature 275 °C; sheath gas flow rate 12 L/min; capillary voltage 3500 V, nozzle voltage 250 V (positive mode)/1000 V (negative mode). The following settings were selected for recording the drift times using the IM cell: frame rate 1 frame/s; IM transient rate 16 IM transients/frame; max drift time 60 ms; trap fill time 3900 µs; trap release time 250 µs; multiplexing pulse sequence length 4 bit. Deviating from the tuning values, the following parameters were changed manually: drift tube entrance 1574 V; drift tube exit voltage 224 V; rear funnel entrance voltage 217.5 V; rear funnel exit voltage 45 V [19,24]. Nitrogen was used as drift gas, which was set to a pressure of approx. 3.95 Torr. Agilent Technologies ESI tune mix was regularly infused as calibrant to calculate the collision cross section (CCS) values from the drift times. All samples were measured in a randomized manner to avoid bias as much as possible. In addition, a quality control (QC) sample was injected every 10 measurements to check the stability of the analytical system. The QC sample consisted of aliquots of all sample extracts from a batch. In addition, targeted MS/MS spectra were recorded from some selected samples at 10, 20, 40 and 60 eV to identify relevant compounds.

### 2.5. Data Processing and Multivariate Data Analysis

The data were first demultiplexed using the PNNL PreProcessor software (version 2020.03.23, publicly available from https://pnnl-comp-mass-spec.github.io/PNNL-PreProcessor accessed on 29 October 2022) with these parameters: demultiplexing checked; chromatography (moving average) 3; moving average smoothing checked; *m*/*z* not used; drift 3; chromatography/infusion 3; signal intensity lower threshold 20 counts; remove spikes checked; saturation repair not checked. Subsequently, the data sets were imported into the MassHunter IMS-MS Browser software (version 10.0, Agilent Technologies) to calculate the CCS values using the single field calibration tool. Feature finding was performed with MassHunter Mass Profiler software (version 10.0, Agilent Technologies) with the following parameters: restrict RT to 0.0–23.0 min; ion intensity 150.0 counts; isotope model common organic (no halogens); limit charge states to a range of 1–2; report single-ion features with charge state z = 1; RT tolerance = 10.0% + 0.50 min; DT tolerance = 1.5%; tolerance = 20.0 ppm + 2.0 mDa; Q-Score 70.0. The formation of adducts was not considered, as this option is currently not supported by the software. If it turned out during the identification of the best marker compounds that a signal was selected twice due to different adducts, only the more intense adduct was used for further evaluation. Since various plant species were analyzed and correspondingly large differences in the samples could be assumed, a feature only had to be detectable in at least 25% of the samples. Both obtained feature tables were exported and transferred to MetaboAnalyst 5.0 software (https://www.metaboanalyst.ca/ accessed on 29 October 2022). Missing values were replaced by the smallest value with which this feature could still be detected in another sample. Subsequently, the data were normalized by sum and autoscaling was performed. Since performing sum normalization can lead to errors depending on the data structure, the intensity distribution of the most relevant marker compounds was also checked using the unnormalized data [25,26,27]. Both PCA and partial least squares data analysis (PLS-DA) score plots were calculated to show correlations and differences in the data. The quality of the PLS-DA model was determined by the Q^2^ values of a leave-one-out cross-validation (LOOCV) in order to rule out possible overfitting. Furthermore, the random forest (RF) algorithm was used to evaluate the quality of the data [28]. To find the best marker substances, a *t*-test and *p*-values or an analysis of variance (ANOVA) as well as the corresponding false discovery rates (FDRs) according to Benjamini-Hochberg were calculated [29]. Furthermore, the Biomarker Analysis tool of MetaboAnalyst was used to calculate receiver operating characteristic (ROC) curves and the areas under the curves (AUCs). For the chemical identification of the best marker compounds, both the MS/MS spectra and, if possible, the CCS values were used. The results obtained were compared with the databases LipidCCS (https://www.metabolomics-shanghai.org/LipidCCS/ accessed on 29 October 2022), MetCCS (https://www.metabolomics-shanghai.org/MetCCS/ accessed on 29 October 2022) as well as LipidMaps (https://www.lipidmaps.org/ accessed on 29 October 2022), HMDB (https://hmdb.ca/ accessed on 29 October 2022) and FooDB (https://foodb.ca/ accessed on 29 October 2022). For MS/MS in in silico fragmentations the software ChemDraw (version 21.0.0.28, PerkinElmer, Waltham, MA, USA) was used.

## 3. Results

**Figure 2:**
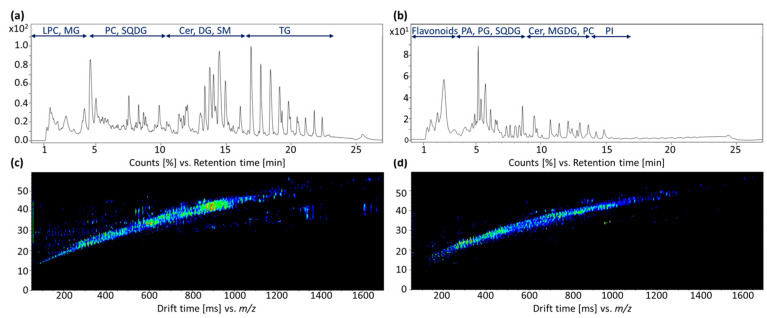
Exemplary data sets of the LC-ESI-IM-QTOF-MS measurements using an *O. vulgare* sample as an example. (**a**) TIC of the measurement in positive ionization mode. (**b**) TIC of the measurement in negative ionization mode. The RT of the chromatographic separation (*x*-axis) is plotted against the signal intensities (*y*-axis). (**c**) Ion mobility spectrum of the sample recorded in positive ionization mode over the entire run time. (**d**) Ion mobility spectrum of the sample recorded in negative ionization mode over the entire run time. The drift time is plotted against the *m*/*z* ratio. The color code reflects the signal intensities.

The number of features detected in a single sample was around 2000–3000 in both positive and negative ionization mode measurements. This magnitude was expected, as shown in previous publications [19,20,21]. Although the fat content of herbs is comparatively low, the composition is highly diverse, which explains this multitude of different compounds that can be seen in the total ion current (TIC) chromatograms (Figure 2a,b). The following classes of compounds were detected in the positive ionization mode: Ceramides (Cers), diacylglycerols (DGs), lysoglycerophosphocholines (LPCs), glycerophosphocholines (PCs), sphingomyelins (SMs), sulfoquinovosyldiacylglycerols (SQDGs) and triacylglycerols (TGs). In the negative mode, especially monogalactosyldiacylglycerols (MGDGs), phosphatidic acids (PAs), phosphatidylglycerols (PG), phosphatidylinositols (PI) and, again, Cers, PCs and SQDGs could be detected. Furthermore, a detailed data analysis showed that not only these typical lipid classes could be detected, but also some flavonoids that eluted early in the measurements performed in negative ionization mode.

In addition to the usual parameters, i.e., retention time (RT), *m*/*z* ratio, and signal intensities, drift times were also recorded using an ion mobility cell. In this way, a further separation of the analytes was achieved, and the number of detectable compounds was increased. Furthermore, an additional identification parameter, the CCS values, was determined. The corresponding sum spectra of the ion mobility measurements are shown in Figure 2c,d, respectively.

### 3.1. Differentiation of the Various Species and Geographical Origins

The feature tables calculated to distinguish the different species and geographical origins, contained 4983 features in the positive ionization mode measurements and 5176 features in the negative ionization mode measurements. Both feature tables had a rather high proportion of missing values, 46% and 48%, respectively, as is often the case for non-targeted measurements with high-resolution mass spectrometers. The proportion of missing values can be reduced by using percentage limits that determine how often a feature must be detected in the analyzed samples. Considering the large diversity between samples due to different geographical origins and different species, this limit was set at 25% to capture most of the observed diversity and to detect metabolites that are present only in subsets of samples. Since missing values can be challenging for some multivariate evaluation methods, they were replaced with the smallest still detectable values to avoid bias in the data structure [30].

**Figure 3:**
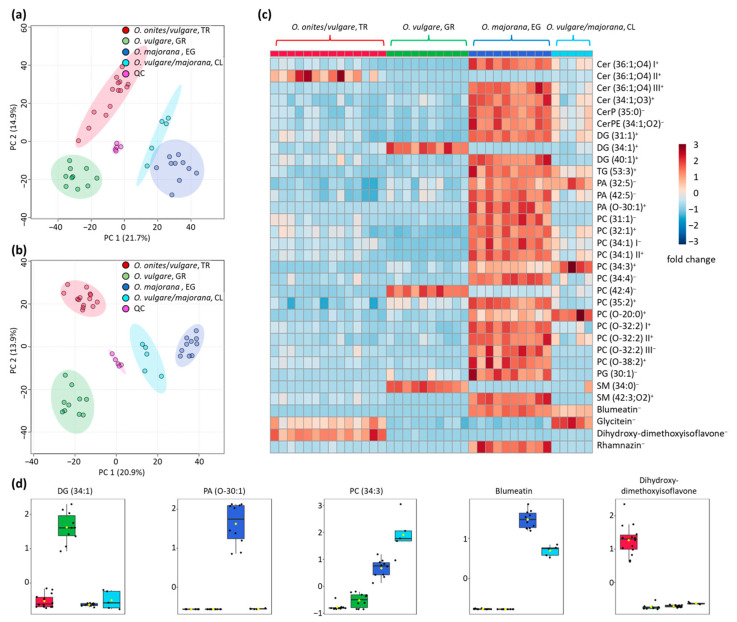
Differentiation of the various species and geographical origins using the LC-ESI-IM-QTOF datasets. (**a**) PCA score plot of measurements in positive ionization mode. (**b**) PCA score plot of measurements in negative ionization mode. (**c**) Heat map of the identified marker compounds that show a dependency on the geographical origin or are suitable for distinguishing the different species. The “+” and “−” signs indicate in which ionization polarity the signals were identified. All marker compounds shown here have an FDR < 0.001 and are therefore present with highly significant signal differences in the various sample groups. (**d**) Concentration distributions of a few selected compounds that are particularly well suited for distinguishing between the different sample groups (red—*O. onites*/*vulgare*, TR; green—*O. vulgare*, GR; dark blue—*O. majorana*, EG; light blue—*O. vulgare*/*majorana*, CL).

First, PCA score plots were calculated to identify potential outliers and to be able to assess the repeatability of the measurements (Figure 3a,b). The QC samples prepared from mixtures of all samples, overlap in the center of the score plots, indicating good repeatability. Furthermore, the score plots show good separation between sample groups for both positive and negative ionization mode measurements, indicating differences between the different sample groups. The *O. majorana* samples from Egypt are relatively close to the putative oregano samples from Chile. The latter were genetically identified as mixtures of *O. vulgare* and *O. majorana*, which was also confirmed by our NMR analysis [10]. Therefore, it was assumed that this group of samples was not authentic, i.e., mislabeled.

The Q^2^ values were calculated using PLS-DA and LOOCV. For the positive ionization mode measurements, the Q^2^ value was 0.94 for five principal components and 0.96 for the negative ionization mode, and also for five components. Thus, both Q^2^ values indicate very significant differences between the sample groups and overfitting can be ruled out. When the RF algorithm was used, one sample in the positive data set was incorrectly assigned. This was one of the samples from Chile that was classified with the *O. majorana* samples from Egypt. In the negative ionization mode, an accuracy of 100% was achieved using the RF algorithm. All samples were correctly assigned, including the samples from Chile, although these were underrepresented leading to a class imbalance, which can be problematic for RF calculations. However, the differences between sample groups were large enough that unambiguous assignment was possible despite this class imbalance.

In total, by calculating an ANOVA and the FDRs, 3327 features were detected in the positive ion mode measurements and 1853 features were detected in the negative ion mode measurements, showing significantly different intensities (FDRs < 0.05) between the sample groups. MS/MS spectra of the most significant 80 signals were recorded for each ionization mode (Table A1 in Appendix A). However, due to the lack of entries in databases such as HMDB, FooDB and LipidMaps, only limited identification of some compounds was possible, or the substances could not be confidently elucidated using high-resolution mass and MS/MS spectra. The identification of the substances was also supported by employing CCS values and comparison with databases. If an entry was available, the deviation was in most cases <2% (Table A1 in Appendix A). Considering that the use of ion mobility cells is still relatively new, there is also a lack of reference data. This applies in particular to the substance class of the ceramides. In addition, the resolution of the currently available ion mobility spectrometers is often not yet sufficient for a reliable identification, which is why we report ranges for values taken from the database. Nevertheless, with increasing resolution of ion mobility cells and improved databases, better assignments can be expected in the coming years [31].

Most of the significant marker compounds were conspicuous, particularly because they were present in elevated concentrations in the marjoram samples from Egypt (Figure 3c). Nevertheless, some other marker compounds could be identified that were detected in higher concentrations in the other sample groups by partially excluding the marjoram samples from the data analysis. A selection of particularly suitable compounds for the discrimination of the different sample groups is shown in Figure 3d. These few analytes are already sufficient to distinguish the different sample groups with an accuracy of 100%.

**Figure 4:**
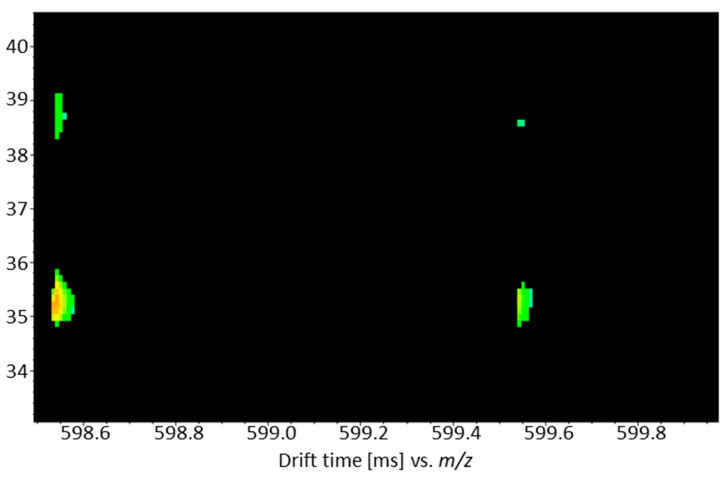
Drift spectra of Cer (36:1;O4) I+ and II+ from a QC sample at an *m*/*z* of 598.54388 and a retention time of 9.3 min. By using an ion mobility cell, the splitting of the signal becomes clear from the different drift times on the *y*-axis. In addition to the [M + H]^+^ signals, further isotope signals are also shown on the *x*-axis.

Some of the compounds were identified more than once, either because they could be detected in both positive and negative ionization mode (PC 34:1 I+ and II−; Table A1 in Appendix A) or because they represent isomers which were separated by liquid chromatography (PC (O-32:2) I+ and II+; Table A1 in Appendix A). Consequently, these are listed several times in Figure 3c and marked with Roman numerals. The relative distributions of signal intensities of such compounds are also quite similar between the different sample groups, either because the measurements at different polarities detected the same compound or relatively structurally similar isomers that presumably perform similar functions in organisms. However, these assumptions are not yet fully understood, as the separation of lipid isomers is often a particular challenge and has only been tackled in recent years thanks to the introduction of ion mobility cells and improvements in chromatographic phases [32]. Furthermore, there are also exceptions, where the distribution of similar features differs (e.g., Cer (36:1;O4) II+ and Cer (36:1;O4) I+ and III+). While the signal intensities of Cer (36:1;O4) II+ were highest in the group of *O. onites/vulgare* samples from Turkey, the other two isomers showed the highest signal intensities in the group of *O. majorana* samples from Egypt. All signals were detected in the positive ionization mode with a relatively identical *m*/*z* of 598.5388 and 598.5389, respectively. In addition, the signals from Cer (36:1;O4) I+ and II+ showed the same retention time at 9.3 min. Nevertheless, they could be distinguished based on their different drift times (Figure 4). Consequently, the differentiation of these signals results from the use of the ion mobility cell and would probably not have been noticed with a normal LC-ESI-QTOF device.

### 3.2. Analysis of Oregano and Marjoram Mixtures

**Figure 5:**
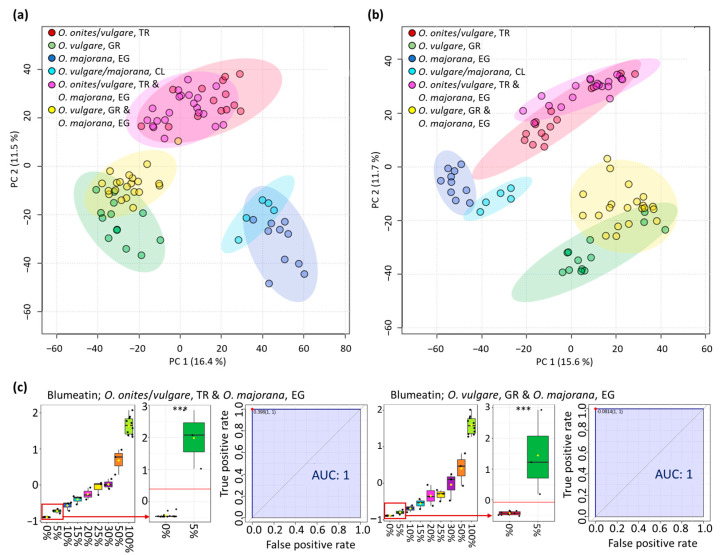
Data analysis of the oregano and marjoram mixtures. (**a**) PCA score plot of measurements carried out in positive ionization mode. (**b**) PCA score plot of measurements carried out in negative ionization mode. (**c**) Concentration distribution of blumeatin in the different mixing ratios. Shown are the normalized signal intensities according to the proportion of marjoram added, as well as the results of the ROC analyses and the AUC values of the pure samples compared to the samples containing 5% marjoram. The number of asterisks reflects the significance of the p-values of the corresponding ROC analyses, which are highly significant (*** *p*-value ≤ 0.001).

To assess whether it is possible to detect additions of marjoram to oregano, mixtures of three *O. onites*/*vulgare* samples from Turkey and three *O. vulgare* samples from Greece with *O. majorana* samples in the ratios of 95/5; 90/10; 85/15; 80/20; 75/25; 70/30; 50/50 were prepared. First, PCA score plots were calculated for the datasets of the two ionization modes (Figure 5a,b). The samples of the mixtures cluster according to their original species (*O. onites*/*vulgare*, TR and *O. vulgare*, GR) and initially show no clear deviations that indicate an admixture of oregano. For an additional analysis, the Pattern Hunter tool of the MetaboAnalyst software was used to look for features present at increasing or decreasing signal intensities for mixtures of increasing *O. majorana* content. Some promising features could be extracted from the measurements in the positive as well as in the negative ionization mode. However, most of them only allowed differentiation of sample groups from marjoram contents of >10%. Finally, based on the measurements in the negative ionization mode, one signal could also be extracted in which admixtures of marjoram in the magnitude of ≥5% were detectable.

This feature has an *m/z* of 301.0717, a retention time of 1.6 min and a CCS value of 172.23 Å^2^ and was later identified as blumeatin (see Section 3.3). The same signal had previously been identified in Section 3.1 (Figure 3d). Using ROC analyses, the performance of this marker compound was assessed by comparing the pure samples with the samples containing 5% marjoram. The AUC values were 1 in each case, indicating that the admixtures were correctly identified with an accuracy of 100%. The respective *p*-values were <0.001, so that a highly significant distinction between the sample groups can be assumed.

### 3.3. Identification of Blumeatin as Marker Compound

Blumeatin (IUPAC: 2-(3,5-dihydroxyphenyl)-5-hydroxy-7-methoxy-3,4-dihydro-2H-1-benzopyran-4-one; CAS: 118024-26-3) was identified in several steps: Using a database search at the HMDB based on the *m*/*z* of 301.0717, 74 possible compounds were initially suggested (molecular weight tolerance ±0.005 Da, status March 2023). This proportion could be reduced to 17 possible substances based on the measured CCS value of 172.23 Å^2^ (collision cross section tolerance ± 3% and the AllCCS prediction method [33]). All remaining proposals were [M−H]^−^-adducts with a molecular formula of C_16_H_14_O_6_, which were further narrowed down based on their MS/MS spectra deposited in HMDB and literature research.

The literature search revealed that hesperetin had already been detected in *O. majorana*, which is why hesperetin was measured as reference standard [34]. Furthermore, the compounds blumeatin, divanillin and porric acid A came into question, since the MS/MS spectra deposited in the HMDB were relatively similar to those of the compound sought. However, it must be noted that some of the MS/MS spectra that are stored in databases are often based on in silico fragmentations and that there may be device-specific or method-specific deviations. Blumeatin and divanillin could both be purchased as reference standards and thus also measured. As far as we know, porric acid A is currently not available as reference substance. Therefore, an attempt was made to extract porric acid A from *Allium porrum* [35].

**Figure 6:**
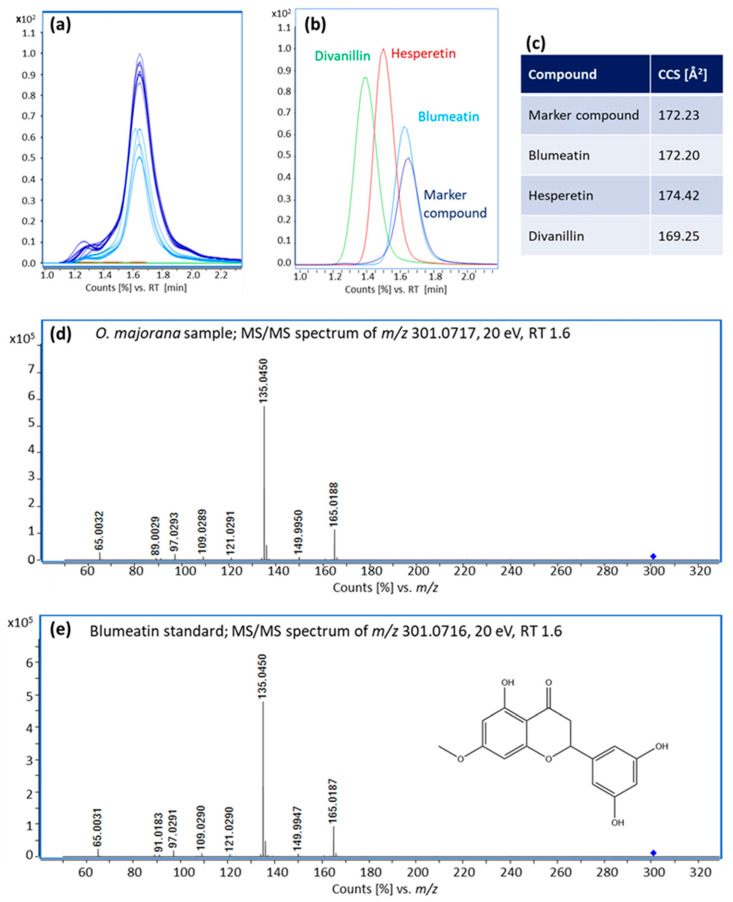
Identification of blumeatin as a marker compound to detect admixtures of marjoram in oregano batches. (**a**) Extracted ion chromatograms (EIC) of the relevant signal from *m*/*z* 301.0717 in the measured oregano and marjoram samples. The measurements of the *O. onites*/*vulgare* samples from Turkey are shown in red, and the measurements of the *O. vulgare* samples from Greece are shown in green. In both sample groups, this marker compound was not detectable. The *O. majorana* samples from Egypt are shown in dark blue and the *O. vulgare* samples from Chile, which according to the NGS analysis also contain marjoram, are shown in light blue. In these two sample groups, the signal is present with significant signal intensities. (**b**) EICs of the signal of *m*/*z* 301.0717 in a marjoram sample and from the standards of blumeatin, hesperetin and divanillin. (**c**) Table of the determined CCS values based on the marjoram sample and the standards of blumeatin, hesperetin and divanillin. (**d**,**e**) MS/MS spectra of the signal of *m*/*z* 301.0717 recorded at 20 eV from a marjoram sample and the blumeatin standard.

**Figure A1:**
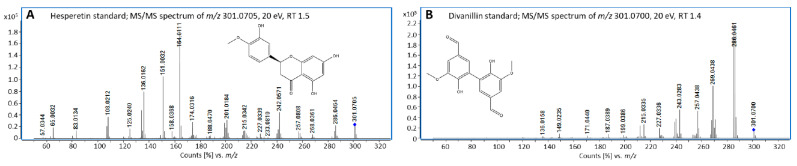
MS/MS spectra of (**A**) hesperetin standard and (**B**) divanillin standard.

The suspicion that it might be porric acid A was not confirmed, as no corresponding signal was detectable in the *A. porrum* extract. Since divanillin and hesperetin showed different retention times, MS/MS spectra and CCS values, they could not be the marker compound either. Instead, the marker compound could be identified as blumeatin using the appropriate reference standard. Besides matching retention times, the identification of blumeatin was also confirmed by MS/MS spectra (Figure A1 in Appendix A) and CCS values (Figure 6). By ensuring correct identification against a reference standard, a structure identification at the highest level 1 (confirmed structure by reference standard) could be achieved according to the proposals of the Metabolomics Standards Initiative [36].

To date, relatively little is known about blumeatin. It is a flavonoid that has been found mainly in *Blumea balsamifera* (Sambong) and has antioxidant, anticarcinogenic and anti-inflammatory effects [37,38]. To our knowledge, it has not yet been detected in marjoram. However, other working groups were able to detect structurally very similar compounds in marjoram [34,39].

To be able to assess whether blumeatin is also present in other plant species used for the adulteration of oregano, three sample extracts each from *O. europaea*, *T. serpyllum* and *T. vulgaris*, *Saliva* ssp. (*S. officinalis* and *S. apiana*), *M. piperita* as well as *M. communis* were measured. The selection of these plant species was based on the results of the European Commission’s report [5]. In the samples containing sage, peppermint, and *T. vulgaris*, signals with an *m*/*z* of 301.0717 could sometimes be detected, but with comparatively low sensitivity and a different retention time. In addition, these signals showed other MS/MS fragments, so they cannot be blumeatin. From these results, it can be concluded that blumeatin is probably a unique marker for marjoram addition, although this assumption certainly needs to be verified on a larger number of samples. A device with an ion mobility cell is not required for this, performing measurements in multiple reaction monitoring (MRM) mode with a LC-ESI-QQQ instrument should be sufficient for this task. In this way, the costs for the analyses can be significantly reduced, since on the one hand LC-ESI-QQQ instruments are relatively widespread and on the other hand comparatively inexpensive to purchase and maintain, so that transfer to routine analysis is relatively easy. In addition, these devices are very sensitive, so it should be possible to further lower the detection limits to meet ESA and the Codex Alimentarius Committee requirements. So far, however, there is no knowledge about concentration levels and variation ranges of blumeatin in marjoram samples. Thus, the analyses should also be extended to a larger data set to include possible influencing factors such as the country of cultivation or the year of harvest.

## 4. Conclusions

Using a high-resolution LC-ESI-IM-QTOF instrument, a non-targeted approach was initially followed to distinguish oregano and marjoram samples of different geographical origins. Measurements were performed in both positive and negative ionization modes. Both data sets provided excellent classification results, in particular, the measurements in negative ionization mode achieved an accuracy of 100% using the RF algorithm. The major marker compounds were identified using MS/MS data and CCS values. It turned out that only a few marker compounds are necessary to separate the different sample groups. Depending on the chromatographic column and the extraction method used, these were mainly lipophilic compounds such as Cers, phospholipids and DGs. Subsequently, mixed samples of oregano and marjoram were analyzed. Although a relatively large number of compounds were suitable for detecting an addition of marjoram >10%, only one compound was suitable for detecting marjoram proportions of the order of 5%. This analyte was identified as blumeatin using a reference standard. Together with arbutin, which we also identified as a suitable marker in a previous study using NMR spectroscopy, a simple, targeted method could be developed, e.g., by means of an LC-ESI-QQQ device, to detect adulterations of oregano with marjoram relatively easily and inexpensively [10]. LC-ESI-QQQ instruments also have the advantage of being more sensitive than LC-ESI-QTOF platforms, especially when using MRM strategies, so that the detection limit of majoram could potentially be lowered to below 5% to meet Codex Alimentarius and ESA requirements.

## Data Availability

The data presented in this study are available on request from the corresponding authors. The data are not publicly available due to privacy.

## Appendix A

The appendix provides further details about the identified metabolites and their CCS values as well as their mass spectrometry parameters. In addition, the MS/MS spectra of the hesperetin and divanillin standard are shown there.

**Table A1:** Identified key metabolites and their ion mobility and mass spectrometry parameters.

Tentative Compound	Proposed Formula	RT [min]	Ionization Mode	Adduct	*m/z* Calculated [Da]	*m/z* Measured [Da]	Error [ppm]	Relevant Fragments	CCS Value Calculated [Å2] *	CCS Value Measured [Å2]	Delta	FDR
Ceramides												
Cer (36:1;O4)	C_36_H_71_NO_5_	9.3	+	[M+H]+	598.5405	598.5388	−2.85	580.53; 562.52	/	262.5	/	3.4×10^−19^
Cer (36:1;O4)	C_36_H_71_NO_5_	9.3	+	[M+H]+	598.5405	598.5388	−2.85	580.53; 562.52	/	287.7	/	4.7×10^−20^
Cer (36:1;O4)	C_36_H_71_NO_5_	10.2	+	[M+H]+	598.5405	598.5389	−2.68	580.53; 562.52	/	262.5	/	6.1×10^−19^
Cer (34:1;O3)	C_34_H_67_NO_4_	8.6	+	[M+H]+	554.5143	554.5122	−3.77	536.50; 518.49	/	255.7	/	5.5×10^−15^
CerP (35:0)	C_35_H_72_NO_6_P	9.8	-	[M-H]-	632.5024	632.4998	−4.18	614.52; 596.52; 152.99	/	260.2	/	7.9×10^−18^
CerPE (34:1;O2)	C_36_H_73_N_2_O_6_P	9.7	-	[M-H]-	659.5133	659.5173	5.98	641.50; 623.46; 152.99	258.5–259.1	264.1	1.9–2.2	2.2×10^−16^
Glycerolipids												
DG (31:1)	C_34_H_64_O_5_	8.0	+	[M+NH4]+	570.5092	570.5081	−1.99	/	252.2–252.3	257.5	2.1	1.3×10^−16^
DG (34:1)	C_39_H_68_O_5_	12.4	+	[M+H]+	617.5115	617.5129	2.28	/	259.8–263.4	259.9	0.0–1.3	3.5×10^−18^
DG (40:1)	C_43_H_82_O_5_	14.0	+	[M+NH4]+	696.6501	696.6533	4.79	/	282.9	284.6	0.6	1.4×10^−16^
TG (53:3)	C_56_H_102_O_6_	19.6	+	[M+NH4]+	888.8015	888.8023	0.96	/	320.1–322.8	337.9	4.7–5.5	2.1×10^−17^
Phospholipids												
PA (32:5)	C_35_H_59_O_8_P	4.8	-	[M+HCOOH-H]-	683.3930	683.3977	7.43	152.99	250.5–251.3	253.4	0.8–1.2	6.1×10^−15^
PA (42:5)	C_45_H_79_O_8_P	8.5	-	[M-H]-	777.5440	777.5442	0.28	309.28; 152.99	276.6–277.2	284.2	2.5–2.7	4.3×10^−15^
PA (O-30:1)	C_33_H_65_O_7_P	4.9	+	[M+H]+	605.4564	605.4597	5.40	/	254.3	248.2	2.4	2.0×10^−18^
PC (31:1)	C_39_H_76_NO_8_P	7.5	-	[M-H]-	716.5236	716.5264	3.93	255.23; 152.99	272.8–273.7	276.6	1.1–1.4	5.1×10^−17^
PC (32:1)	C_40_H_78_NO_8_P	8.9	+	[M+H]+	732.5538	732.5529	−1.20	184.07	281.9	284.1	0.7	6.3×10^−16^
PC (34:1)	C_42_H_82_NO_8_P	8.8	-	[M-H]-	758.5705	758.5746	5.36	283.26; 253.25; 152.99	281.9	284.5	0.9	5.1×10^−17^
PC (34:1)	C_42_H_82_NO_8_P	10.2	+	[M+H]+	760.5851	760.5840	−1.42	184.07	288.1–288.2	287.8	0.1	1.5×10^−18^
PC (34:3)	C_42_H_78_NO_8_P	8.7	+	[M+H]+	756.5538	756.5566	3.73	184.07	283.3–285.4	285.5	0.1–0.8	3.0×10^−15^
PC (34:4)	C_42_H_76_NO_8_P	8.5	-	[M-H]-	752.5236	752.5254	2.42	152.99	276.7–279.2	280.0	0.3–1.2	4.4×10^−18^
PC (42:4)	C_50_H_92_NO_8_P	12.3	-	[M-H]-	864.6488	864.6504	1.87	152.99	299.0–299.8	302.2	0.8–1.0	1.2×10^−20^
PC (35:2)	C_43_H_82_NO_8_P	9.8	+	[M+H]+	772.5851	772.5872	2.75	184.07	287.9–289.1	289.2	0.0–0.5	4.9×10^−15^
PC (O-20:0)	C_28_H_58_NO_7_P	6.2	+	[M+H]+	552.4024	552.4009	−2.66	184.07	243.7–245.0	245.0	0.0–0.5	6.7×10^−26^
PC (O-32:2)	C_40_H_78_NO_7_P	8.1	+	[M+H]+	716.5589	716.5607	2.56	/	278.2–279.0	281.9	1.0–1.3	2.8×10^−20^
PC (O-32:2)	C_40_H_78_NO_7_P	8.6	+	[M+H]+	716.5589	716.5637	6.75	184.07	278.2–279.0	283.3	1.5–1.8	1.7×10^−15^
PC (O-32:2)	C_40_H_78_NO_7_P	8.3	-	[M-H]-	714.5443	714.5485	5.85	152.99	273.2–274.0	276.7	1.0–1.3	1.5×10^−16^
PC (O-38:2)	C_46_H_90_NO_7_P	5.4	+	[M+H]+	800.6528	800.6571	5.42	184.07	296.0–297.8	297.7	0.0–0.6	1.1×10^−14^
PG (30:1)	C_36_H69O_10_P	5.5	-	[M-H]-	691.4556	691.4528	−3.98	255.23; 225.18	256.6	261.9	3.2	9.2×10^−15^
SM (34:0)	C_39_H_82_N_2_O_6_P	12.7	-	[M-H]-	704.5838	704.5845	1.03	668.61; 152.99	/	276.2	/	4.2×10^−17^
SM (42:3;O2)	C_47_H_91_N_2_O_6_P	12.5	+	[M+NH4]+	828.6953	828.6943	−1.23	184.07	300.7	304.4	1.2	1.9×10^−20^
Flavonoids												
Blumeatin	C_16_H_14_O_6_	1.6	-	[M-H]-	301.0718	301.0717	0.00	165.02; 135.05	170.2	172.2	1.2	2.1×10^−29^
Glycitein	C_16_H_12_O_5_	2.1	-	[M-H]-	283.0612	283.0597	−5.27	268.04; 239.03	165.3	166.0	0.4	2.6×10^−16^
2’,7-Dihydroxy-4’,5’-dimethox-yisoflavone	C_17_H_14_O_6_	1.3	-	[M-H]-	313.0718	313.0696	−6.88	161.02	171.9	175.2	1.9	7.9×10^−18^
Rhamnazin	C_17_H_14_O_7_	1.6	-	[M-H]-	329.0667	329.0648	−5.68	314.04; 299.01; 271.02	/	176.9	/	1.6×10^−18^

Abbreviations: Cer, ceramide; CerP, ceramidephosphate; CerPE, ceramide phosphoethanolamine; DG, diacylglycerol; PA, phosphatidic acid; PC, glycerophosphocholine; SM, sphingomyelin. * LipidCCS (https://www.metabolomics-shanghai.org/LipidCCS/) and MetCCS (https://www.metabolomics-shanghai.org/MetCCS/), HMDB (https://hmdb.ca/), FooDB (https://foodb.ca/) accessed on 29 October 2022.
